# A Randomized Controlled Trial to Assess the Impact of Preoperative Stoma Simulation on Anxiety and Postoperative Adaptation: A Study Protocol

**DOI:** 10.1002/nop2.70682

**Published:** 2026-07-07

**Authors:** Hatice Merve Alptekin, Yasemin Özhanli

**Affiliations:** ^1^ Department of Surgical Nursing, Faculty of Health Sciences Kocaeli University Izmit Kocaeli Türkiye

**Keywords:** adaptation, anxiety, colorectal cancer, simulation, stoma

## Abstract

**Aim:**

This study aims to evaluate the effects of preoperative stoma simulation on preoperative state anxiety and postoperative stoma adaptation among individuals scheduled for stoma surgery.

**Design:**

This study is designed as a two‐arm, parallel‐group randomized controlled trial.

**Methods:**

A total of 52 patients scheduled for stoma surgery will be randomly allocated to either the intervention group (*n* = 26) or the control group (*n* = 26). The intervention group will receive a structured preoperative stoma simulation in addition to standard care, whereas the control group will receive standard preoperative care alone. The primary outcome is preoperative state anxiety, which will be measured using the Spielberger State Anxiety Inventory 24 h before surgery and on the morning of surgery. The secondary outcome is postoperative stoma adaptation, which will be assessed at discharge and 1 month after surgery using the Ostomy Adjustment Inventory. Data will be collected through face‐to‐face interviews. Outcome assessments will be conducted by a blinded assessor, and statistical analyses will be performed by a blinded statistician.

**Results:**

As this manuscript presents the study protocol, the results are not yet available. The findings are expected to provide evidence regarding the effectiveness of preoperative stoma simulation in reducing anxiety and improving postoperative adaptation.

**Conclusion:**

This trial is expected to provide evidence regarding the effectiveness of preoperative stoma simulation on preoperative state anxiety and postoperative stoma adaptation and may contribute to the development of evidence‐based nursing interventions for individuals undergoing stoma surgery.

**No Patient or Public Contribution:**

Patients or the public were not involved in the design, conduct, reporting, or dissemination plans of this research.

**Trial Registration:**

ClinicalTrials.gov Identifier: NCT06737887. Registered on December 11, 2024

## 1 Introduction

Colorectal cancer (CRC) is among the most common malignancies worldwide and ranks third among cancer‐related causes of death (Polat et al. 2021). According to the Global Cancer Statistics Report (Globocan, 2020 edition), there are an estimated 1.9 million new cases of CRC and 930,000 deaths. The incidence rate is highest in men in Australia/New Zealand at 40.6 per 100,000 and lowest in women in several African regions and South Asia at 4.4 per 100,000. The mortality pattern for this cancer type is similar to that of incidence (Sung et al. 2021; Morgan et al. 2023). The burden of CRC is projected to increase by an average of 45% in incidence and mortality by 2040, with the majority of cases in countries with high or very high human development indices (HDIs) (Xi and Xu 2021).

It is estimated that an average of 725,000–1,000,000 people in the U.S. (UOAA 2024) and 13,500 people in the UK (Nazarko 2024) live with a stoma. Stoma surgery, which is frequently used in treating patients with colorectal cancer, contributes significantly to the economy by prolonging life expectancy, improving health outcomes, and improving quality of life (Zewude et al. 2021). Health professionals should support patients' adaptation to the stoma by providing stoma‐related education and skill training, thereby enhancing self‐care knowledge, self‐care performance, and independence in stoma management (Momeni Pour et al. 2023). There is evidence that preoperative training can help patients adapt well to the process after surgery, reduce physical and psychological problems, and shorten the duration of hospitalization (Hughes et al. 2020; Brodersen et al. 2023).

## 2 Background

Stoma is a medical and social problem worldwide. Approximately 100,000 patients undergo stoma surgery annually in the United States (Ferrara et al. 2019). Although stoma surgery is considered a therapeutic approach in individuals with colorectal cancer, it causes the loss of voluntary bowel control and privacy, and the unnoticed excretory process becomes visible and noticeable (Ayik et al. 2018; Duluklu and Çelik 2019).

In this surgical intervention, which changes the excretory mechanism direction and the functionality of existing muscles, individuals gain various physiological, psychological, and social experiences (Liu et al. 2024). These experiences, starting from the preoperative period, affect the postoperative colostomy adaptation process and the general quality of life and lead to positive or negative outcomes (Ayik et al. 2018; Duluklu and Çelik 2019). Therefore, it is important to determine the appropriate nursing interventions for patients with colostomy to cope with and adapt to current physical changes.

Despite the development of surgical intervention techniques and disposable care products for stoma care, stoma‐related complications account for an average of two‐thirds of cases (Ferrara et al. 2019). In the development of complications, stoma type, region, failure to mark the surgical site, failure to determine the region well, emergency surgery, inadequate preoperative preparation and postoperative care, failure to manage anxiety, and other comorbid conditions affect the patient (Chan et al. 2023; Altintas et al. 2025).

Individuals with a stoma require education and counselling both before and after surgery to effectively manage stoma‐related care and successfully adapt to life with a stoma (Akbayrak and Uslu 2026). Patient education should start with the first admission to the clinic; accurate information about the procedures should be conveyed to patients and their relatives understandably. It was determined that 36% of patients scheduled for stoma surgery did not receive preoperative marking, and 6% developed parastomal hernia complications (Kozan and Gültekin 2018). Marking the area before stoma surgery reduces the likelihood of potential stoma problems such as leakage, bag insertion difficulties, associated skin irritation and pain, and improves the quality of life of patients in the postoperative period (Gök et al. 2019).

On the basis of this information, simulating this artificial organ while the patient spends a day with a stoma bag before elective surgery, where he/she performs the actions of sitting, getting up, walking, going to the toilet, dressing, and communicating, can regulate the patient's anxiety, allow him/her to ask the nurse for help with difficulties, and improve the postoperative adaptation process.

The hypotheses of the study are as follows:

*Stoma simulation intervention reduces patients' preoperative anxiety*.

*Stoma simulation intervention increases patients' adaptation to the stoma after surgery*.

## 3 Method

### 3.1 Trial Status

This trial was registered at ClinicalTrials.gov (Registration number: NCT06737887) on December 11, 2024. The current protocol version is Version 1.0 (January 2, 2027). Participant recruitment is planned to begin on January 2, 2027, and it is expected to be completed by January 2, 2028.

### 3.2 Design

**FIGURE 1:**
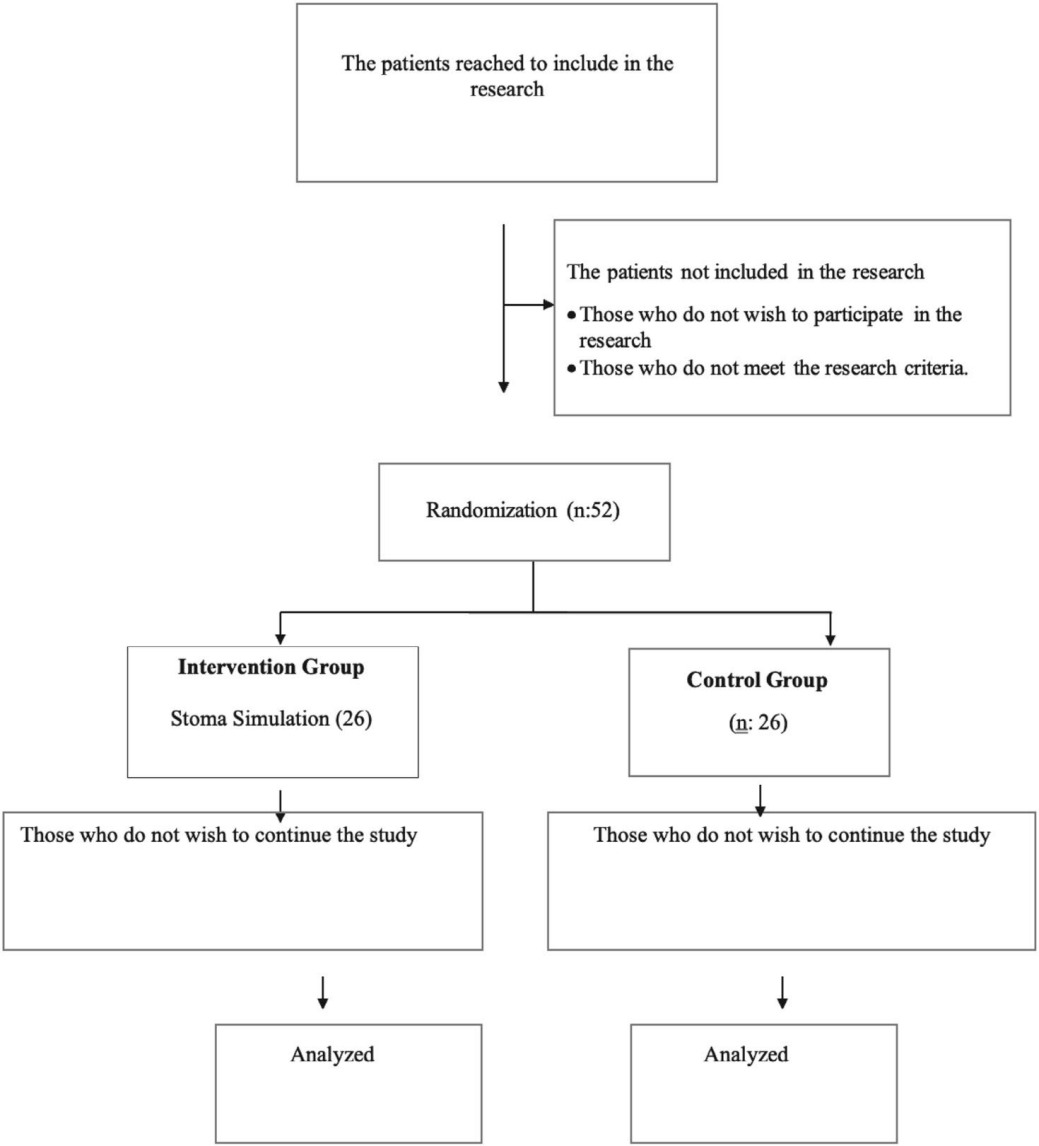
CONSORT flowchart.

This study will be a two‐arm, parallel‐group randomized controlled trial with blinded outcome assessment and blinded data analysis in patients scheduled for stoma surgery. The aim of this study is to evaluate the effects of preoperative stoma simulation on patients' anxiety levels and postoperative stoma adaptation. Patients who meet the inclusion criteria and agree to participate in the study will be randomized to the stoma simulation (intervention group) or control group. The application will be performed 1 day before surgery. Patients will be evaluated on the morning of surgery, at discharge, and 1 month later. The primary outcome (anxiety) and secondary outcome (stoma adaptation) will be assessed at different time points. All phases of the study followed the Consolidated Standards for Reporting Trials (CONSORT) standards and the SPIRIT Checklist (Boutron et al. 2017). The planned participant flow of the study is presented in the CONSORT flowchart (Figure 1).

### 3.3 Inclusion Procedure

Participants will be recruited from an advanced medical faculty hospital that accepts patients from different regions of the country. Patients scheduled for stoma surgery will be identified by the responsible nurse and stoma care nurse working in the general surgery service of the hospital. The nurses will bring the researchers together with the patients who meet the study criteria and are scheduled for stoma surgery. Patients will be informed about the study by the researchers. Written informed consent will be obtained from the patients willing to participate in the study. At the same time, the researcher's contact information will be left with the patients, and they will be able to contact them at any time in case of difficulties they encounter.

### 3.4 Participants'

#### 3.4.1 Inclusion Criteria

The following patients were included in the study:

- Those who are between the ages of 18 and 65.
- Who can speak Turkish.
- Who can read and write.
- Who is conscious.
- Who is willing to participate in the study?

#### 3.4.2 Exclusion Criteria

The following patients were excluded from the study:

- Those who are impaired in speech or hearing.
- Those with urostomy surgery.
- Those who need intensive care after surgery.
- Those whose stoma was closed during the study.
- Those who have had complications in the peristomal area.
- Those who had undergone stoma surgery before.
- Because conditions or medications that could independently affect anxiety levels, which is the primary outcome of the study, individuals with a diagnosed psychiatric disorder or who are currently taking psychiatric medication will not be included in the study.

### 3.5 Sample

The study will be conducted in an advanced hospital where approximately 9–12 stoma surgeries are performed monthly. The sample size was calculated using G*Power 3.1.9.2. The calculation was based on the primary outcome of the study, state anxiety. The effect size was calculated using the State Anxiety Inventory scores reported by Akbayrak and Uslu (2026), who evaluated the effect of preoperative stoma education including a wearable dummy stoma on postoperative stoma‐related anxiety, adjustment, and self‐efficacy. In that study, the mean State Anxiety Inventory scores were 36.24 ± 8.15 in the experimental group and 48.16 ± 10.14 in the control group. Based on these values, the effect size was calculated as *d* = 1.29. Using a two‐tailed independent‐samples *t* test, *α* = 0.05, power (1 − *β*) = 0.99, effect size = 1.29, and an allocation ratio of 1:1, the minimum required sample size was calculated as 46 participants, with 23 participants in each group. Considering a possible 10% attrition rate during follow‐up, including stoma closure, mortality, or participant withdrawal, the total planned sample size was increased to 52 participants, with 26 participants in each group.

### 3.6 Randomization

Participants who meet the eligibility criteria and provide written informed consent will be randomly assigned to either the intervention group (*n* = 26) or the control group (*n* = 26) in a 1:1 allocation ratio. A computer‐generated block randomization method with a fixed block size of four will be used to ensure balanced allocation throughout the study.

The randomization sequence will be generated before participant recruitment by an independent researcher who will not be involved in participant recruitment, intervention delivery, outcome assessment, or data analysis.

To ensure allocation concealment, the group assignments will be placed in sequentially numbered, opaque, sealed envelopes prepared by the independent researcher. After participant enrolment and completion of baseline assessments, the intervention researcher will open the next envelope in sequence to determine group allocation. This procedure will be implemented to minimize selection bias and preserve the integrity of the randomization process.

### 3.7 Blinding

Owing to the nature of the intervention, the participants and the researcher performing the intervention cannot be blinded. However, outcome assessments will be performed by an independent researcher who is blinded to group allocation and is not involved in participant recruitment, randomization, or intervention delivery. Participants will be instructed not to disclose their group assignment during outcome assessments. In addition, an independent statistician blinded to group allocation will conduct the statistical analyses. The expert statistician will not be aware of the randomization sequence or group allocation and will not participate in the intervention implementation. The data obtained from the study will be recorded in the SPSS 21 program by the intervention researcher. Before statistical analysis, the intervention and control groups will be coded as Group A and Group B to maintain blinding. The expert who evaluates the study's data will not know which results belong to the intervention or the control group. He/she will share the study results with the researchers, for example, group A and group B results. Therefore, the study will incorporate blinded outcome assessment and blinded data analysis to minimize detection bias.

### 3.8 Intervention

#### 3.8.1 Preparation for Simulation Application

Preparation for simulation implementation is critical to the success of this study. At this stage, preparations are required to implement the simulation effectively. First, the goals and purpose of the simulation should be determined. Stoma simulation aims to enable patients to understand the surgical process and postoperative stoma care, reduce their anxiety levels, and accelerate the postoperative adaptation process.

For this reason, the materials to be used in the simulation will be carefully selected and prepared 1 day before surgery.

**FIGURE 2:**
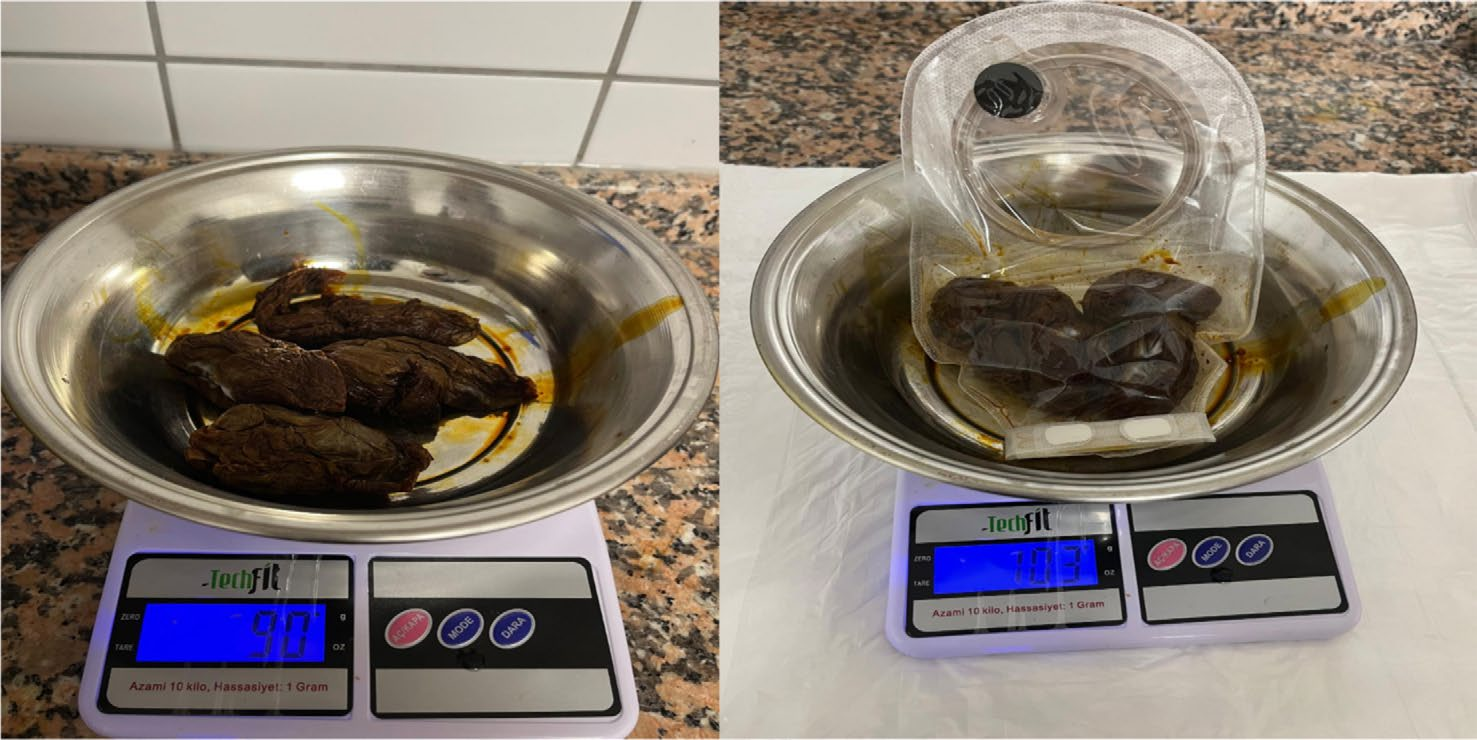
Preparation stages of the stoma simulation.

Since the patients used a two‐piece stoma bag in the long term, this material will be used in the simulation application. In addition, transparent stoma bags will be used because patients use them in the early postoperative period, and they will be familiar with the contents of the stoma. Coloplast products will be used according to the clinical protocol. The ‘Alterna Base Plate’ will be used for the stoma adapter and the ‘Alterna 2‐Piece, Drainable Bag’ will be used for the stoma bag. In addition, ‘Coloplast Paste’ will be used at the mouth of the stoma adapter. Care will be taken to use every product that should be used while providing routine stoma care while the stoma bag is sticking to the patient. This is a good opportunity for patients to see and familiarize themselves with the products used closely. To make the stoma bag realistic, soaked, reshaped napkins and tap water will be placed inside the bag. The amount of water will be 40 cc. The soaked napkins will be weighed with a precision scale, as equal amounts are desired for each patient. The weight of the soaked napkins will be 50 g. In the study aiming at stoma practice, it is seen that only tap water was placed in the bag (Karaveli and Demirarslan 2021). However, in this study, since patients who will open colostomy and ileostomy will be included, the content in the bag will be prepared in a more solid form. For this reason, both napkins and water will be placed in the bag. The weight of the stoma bag is 13 g. The napkins will also be dyed with ‘batikon’ to make the contents of the stoma bag resemble stool. The preparation stages of the stoma simulation are presented in Figure 2. Thus, as the duration of use increases, it will be indistinguishable from a real stoma and a stoma bag. ‘Brava Adhesive Remover, spray’ and ‘Brava Adhesive Remover, wipes’ will also be used when removing the stoma bag. This removes all paste residues and reduces skin peeling and skin damage. It will also increase comfort by reducing the degree of discomfort experienced by patients.

#### 3.8.2 Intervention Group

The stoma care nurse will inform the researcher, who will perform the application of the patients who meet the study criteria. In addition, the stoma care nurse will mark the area where the stoma will be opened with a surgical skin marking pen that is nontoxic, iodine‐free, resistant to rubbing, easy to dry, and suitable for surgical interventions. In addition to routine preoperative care and standard stoma education, patients in the intervention group will receive a stoma simulation intervention. Throughout the study, potential side effects related to the intervention (such as skin irritation, physical discomfort, or psychological distress) will be monitored. If any adverse events occur, the participant will be evaluated by the clinical team, and appropriate support will be provided when necessary. All adverse events will be recorded and reported by the researchers. If serious physical or psychological adverse events related to the intervention are observed, the participant's involvement in the intervention will be discontinued, and the study process will be re‐evaluated by the researchers. For the patients in the intervention group, the researcher will measure their state and trait anxiety levels after informing them about the study and providing written informed consent. A preprepared stoma bag containing wet napkins (information about the contents will be given but not shown to the patients) will be attached to the patient. When the stoma bag is attached to the patient, care will be taken to ensure that it is in the area marked by the stoma nurse. The adapter will be cut into an estimated stoma size, heated by friction of the hands, and adapted to the skin by applying stoma paste. The patient will be asked to put his/her hand on the adapter and apply pressure. Then, the stoma bag is filled with napkins and water, and the adapter will be combined. The patient will be informed that he/she should not hesitate to perform any activity with the stoma bag for 24 h. The patient will be allowed to go to the toilet, eat, sleep, and perform all daily activities with the stoma bag. Brava Adhesive Remover, spray, and Brava Adhesive Remover wipes will be used to clean the adapter adhesives and stoma paste residues on the morning of surgery. The patient's state of anxiety will be measured after the removal of the stoma bag. During the adaptation and removal of the stoma bag to the skin, the steps described in the literature will be followed (American Cancer Society 2019; Kirkland‐Kyhn et al. 2018). It may take time for patients who have had a stoma to adapt. Long‐term follow‐up is necessary for the study results to be more effective. In the literature, evaluating stoma adaptation in patients approximately 1 month after surgery is considered appropriate (Taylan and Aksoy 2021; Zhang et al. 2019; Whiteley et al. 2024). For this reason, stoma adaptations of patients will be evaluated at the time of discharge and 1 month after surgery. If patients have a possible problem with the stoma bag at night before surgery or have questions they want to ask, the information of the 24/7 open phone number where they can contact the researchers will be shared.

#### 3.8.3 Control Group

As in the intervention group, patients who meet the criteria will be reported to the researchers by the stoma care nurse. The researcher will inform patients, and written informed consent will be obtained. One day before surgery, patients' state and trait anxiety levels will be evaluated. In addition, stoma adaptation of the patients will be evaluated at the time of discharge and 1 month after surgery. Patients in the control group will receive routine preoperative care and standard stoma training provided by a clinical nurse and a stoma care nurse. No additional stoma simulation procedure will be performed in this group.

### 3.9 Data Collection

The data of the study will be collected with the ‘Patient Information Form’, ‘Spielberg State‐Trait Anxiety Scale’ and ‘Adaptation Scale for Individuals with Ostomy’ prepared by the researchers in light of the literature. Before stoma surgery, patients who meet the inclusion criteria will be interviewed and informed about the study. Patients who give verbal and written consent to participate in the study will be included. The ‘Patient Information Form’ and ‘Spielberg State‐Trait Anxiety Scale’ will be applied to the patients 24 h before stoma surgery. The ‘Spielberg State Anxiety Scale’ will be applied on the morning of surgery. The ‘Adaptation Scale for Individuals with Ostomy’ will be applied at discharge and 1 month after surgery (Table 1). The questions in the data collection form will be applied with a face‐to‐face interview technique. Each interview is expected to last approximately 15–20 min. Data will be collected in patient rooms to make patients feel comfortable. During the follow‐up to be performed in the first month after surgery, patients are called to the physician's control in accordance with the hospital protocol. After physician control, the researcher will have a face‐to‐face interview with the patients in the stoma nurse's room and evaluate the stoma adaptation.

**TABLE 1:** Participant timeline: Schedule of enrollment, interventions and assessments.^a^

Timepoint^b^	Trial period					
	Enrollment		Post‐randomization			Close out
	−*t*_*i*_ to 0	0	*t*_1_ (24 h before surgery)	*t*_2_ (surgery morning)	*t*_3_ (discharge)	*t*_4_ (1 month ±7 days)
Enrollment						
Eligibility screen	X					
Informed consent	X					
[Baseline demographic data]	X					
Randomization		X				
Intervention/comparator						
[Stoma simulation]^c^			X	X (removal)		
[Control]^d^						
Assessments						
[STAI‐trait anxiety]	X			X		
[STAI‐state anxiety]	X			X		
[Ostomy Adjustment Inventory]					X	X

^a^ Recommended content can be displayed using various schematic formats.

^b^ List target timepoints and acceptable time windows in this row (e.g., 30 ± 3 days).

^c^ Arrow indicates continuous delivery of intervention (e.g., drug).

^d^ Example illustrates delivery of comparator at discrete timepoints (e.g., psychotherapy).

### 3.10 Data Collection Tools

#### 3.10.1 Patient Information Form

This form was prepared by researchers on the basis of the literature (Diniz et al. 2023; Taylan and Aksoy 2021; Xavier et al. 2024). It includes individual factors (control variables) that will affect stoma adaptation. It consists of nine questions, including the patient's age, gender, medical diagnosis, and stoma‐related characteristics, such as placement and type.

#### 3.10.2 Spielberg State‐Trait Anxiety Scale

The scale was developed by Spielberger et al. (1970), and its Turkish validity and reliability study was conducted by Öner and Le Compte (1983). The scale consists of two separate dimensions, namely, state anxiety (STAI‐I) and trait anxiety (STAI‐II), and these dimensions aim to measure the anxiety states of individuals in different contexts. State anxiety measures the transient anxiety that an individual feels at a specific moment and in a specific situation and can be influenced by environmental factors, immediate stressors, or specific events. The anxiety felt by the patient before surgery is assessed with this dimension. The questions aim to understand the level of anxiety, tension and restlessness the person feels in the current situation. Trait anxiety is the individual's level of predisposition to anxiety in general. It is more closely related to an individual's personality traits and refers to a more prolonged state. The fact that a person usually has an anxious personality in daily life is evaluated with this dimension. It aims to understand how the individual reacts to anxiety and stressful situations in general. In this study, the anxiety scale will be used to assess the anxiety levels experienced by individuals who will undergo stoma surgery in a specific situation or continuously (Karaveli Çakır and Özbayır 2018; Öner and Le Compte 1983; Spielberger et al. 1970).

The scale consists of 40 items; 20 items measure state anxiety and the other 20 measure trait anxiety. The scale items are 4‐point Likert‐type. Responses range from ‘Never’ (1) to ‘Very often’ (4). The state anxiety scale includes 10 inverse statements (1, 2, 5, 8, 11, 15, 16, 19, 20) and 10 straight statements, whereas the trait anxiety scale includes 7 inverse statements (21, 26, 27, 30, 33, 36, 39) and 13 straight statements. Inverted statements represent positive emotions, and straight statements represent negative emotions. Although the scores of the inverse statements are evaluated by transforming them, the scores of the straight statements are used directly. Both scales are scored between 20 and 80, and the higher the score is, the higher the anxiety level. In the validity and reliability study, the Cronbach's alpha (*α*) coefficient value was between 0.94 and 0.96 for the STAI‐I and between 0.83 and 0.87 for the STAI‐II (Öner and Le Compte 1983).

#### 3.10.3 Ostomy Adjustment Inventory

The scale was developed by Simmons et al. (2009), and the Turkish validity and reliability study was conducted by Karadağ et al. (2011). It is used to understand the adaptation process experienced by individuals with ostomy after a stoma and to determine in which directions this process should be supported. The scale systematically addresses the factors affecting the adaptation of individuals to their new lifestyles. It is a multidimensional scale that evaluates factors related to patients' activities of daily living, emotional adjustment, psychological adjustment, and social isolation. This study evaluated the adaptation of patients with stoma at discharge and 1 month after opening. The scale consists of 23 items and four subscales. The subdimensions are acceptance, anger, anxiety/worry, and social adjustment. The scale items included acceptance (1, 3, 4, 6, 9, 14, 15, 19, 23), anxiety/worry (12, 13, 17, 20, 21), social adjustment (5, 7, 8, 11), and anger (2 and 10). Items 16, 18 and 22 were not included in any subdimension. The 12 items in the scale (2, 5, 7, 8, 10–13, 16–18, 21) are calculated by reversing. The scale items are 5‐point Likert‐type. The response options range from ‘Strongly disagree’ (0) to ‘Strongly agree’ (4). The lowest score is 0, and the highest score is 92. A high score on the scale indicates that individuals have high stoma adaptation. In the validity and reliability study, the Cronbach's alpha (*α*) coefficient value was 0.874 (Karadağ et al. 2011).

### 3.11 Evaluation of the Data

The data obtained from the study will be analysed using SPSS 21 (Statistical Package for the Social Sciences) software. First, the demographic and clinical characteristics of the participants will be summarized using descriptive statistics (frequencies, percentages, means and standard deviations). To assess baseline comparability between groups, independent‐samples *t* test will be used for normally distributed continuous variables, Mann–Whitney *U* tests for non‐normally distributed continuous variables, and chi‐square tests for categorical variables.

The primary outcome of the study is state anxiety measured using the State Anxiety Inventory. Changes in state anxiety scores between the intervention and control groups over time will be evaluated using repeated‐measures analysis of variance (ANOVA), including group, time, and group‐time interaction effects. The secondary outcome of the study is postoperative stoma adaptation measured using the Ostomy Adjustment Inventory. Changes in stoma adaptation scores between groups and across follow‐up measurements will also be evaluated using repeated‐measures ANOVA.

Age, gender, diagnosis, stoma type, baseline trait anxiety scores and baseline state anxiety scores will be prespecified as potential covariates. Baseline comparability between groups will be assessed, and adjusted analyses will be performed where clinically relevant imbalances are identified.

All primary analyses will be conducted according to the intention‐to‐treat (ITT) principle. Missing outcome data will be handled using multiple imputation methods. In addition, a per‐protocol analysis including participants who complete all planned assessments will be conducted as a sensitivity analysis. A significance level of *p* < 0.05 will be accepted in all analyses.

Every effort will be made to minimize missing data throughout the study. Reasons for missing data, participant withdrawals, mortality, and stoma closure will be documented and reported in detail. Results will be reported with 95% confidence intervals, effect sizes, and *p* values. The clinical significance of the findings will also be emphasized.

### 3.12 Ethical Approach

Ethical approval was obtained from the Kocaeli University Non‐Invasive Clinical Research Ethics Committee (14 Dec 2023 approval number: KÜ GOKAEK‐2023/20.19, project number: 2023/410). Patients will be informed that the answers given and all written information will be kept by the researchers; their answers will remain confidential and used only for scientific purposes. Patients will also be informed that they can leave the study at any time. Owing to the low‐risk nature of the intervention and the single‐center design of the study, an independent data monitoring committee will not be established. All study procedures will be carried out in accordance with the ethical standards of the Declaration of Helsinki; patients included in the study will be informed about the study, and verbal and written consent will be obtained. Patients participating in the study will be asked to sign an informed consent form stating that they have been informed and are volunteers.

## 4 Discussion

In stoma care, some studies have contributed to the professional development of stoma care nurses or nurses with simulation‐based training (Almeida et al. 2021; MacLean and Patton 2024). In addition, stoma simulation is included in the education of nursing students who will be future nurses (Lucena et al. 2023). To increase the empathy of nursing students and evaluate their thoughts about patients with stomas, a stoma simulation was performed by placing a stoma bag in the abdomen of the students, and the results were evaluated (Karaveli and Demirarslan 2021). Studies have shown that simulation is an effective training method. However, studies with patients are limited. Pouresmail et al. (2019) evaluated the effect of a stoma simulation device on patients' postoperative self‐efficacy. Although this training affects self‐care/self‐efficacy because patients already have a stoma after surgery, routine stoma care may also have an effect. As suggested in this study, a physical stoma simulation is needed for patients' first encounter with the stoma. This study protocol aims to contribute to the limited literature on preoperative stoma simulation in patients undergoing stoma surgery.

## 5 Limitations

This protocol has several limitations. First, the demographic characteristics and health status of the patients included in the study may limit the generalizability of the results obtained. In addition, the fact that the data will be collected from a single hospital may also make it difficult to generalize the results. Failure to fully control for external factors (e.g., family support, economic status, etc.) that may affect patients' anxiety levels may also have an impact on the results. Furthermore, excluding patients with a psychiatric diagnosis or those taking psychiatric medication may limit the generalizability of the findings. In addition, participant blinding will not be possible due to the nature of the intervention, which may increase the risk of performance bias. Finally, since it will be a long‐term study requiring follow‐up, patients' stoma may close, they may die, or they may want to drop out of the study during the study period, so there may be missing data.

## 6 Conclusion

This study protocol is expected to provide evidence regarding the potential effects of preoperative stoma simulation on patients' anxiety levels and postoperative stoma adaptation. If effective, preoperative stoma simulation may support patients' psychological preparation for surgery and adaptation to life with a stoma. In addition, the intervention may help patients become more familiar with postoperative stoma care and related equipment before surgery. The findings of this study may contribute to the development of supportive nursing interventions for individuals undergoing stoma surgery.

## Author Contributions

**Hatice Merve Alptekin:** conceptualization, methodology, writing – original draft, project administration, writing – review and editing. **Yasemin Özhanli:** methodology, writing – review and editing.

## Funding

The authors have nothing to report.

## Ethics Statement

Ethical approval was obtained from the Kocaeli University Non‐Invasive Clinical Research Ethics Committee (14 Dec 2023‐approval number: KÜ GOKAEK‐2023/20.19, project number: 2023/410). Patients will be informed that the answers given and all written information will be kept by the researchers; their answers will remain confidential and used only for scientific purposes. Patients will also be informed that they can leave the study at any time. Owing to the low‐risk nature of the intervention and the single‐center design of the study, an independent data monitoring committee will not be established. All study procedures will be carried out in accordance with the ethical standards of the Declaration of Helsinki; patients included in the study will be informed about the study, and verbal and written consent will be obtained. Patients participating in the study will be asked to sign an informed consent form stating that they have been informed and are volunteers. Information about the study was uploaded to the Clinical Trials platform, and a Clinical Trials ID number was obtained. Clinical Trials ID: NCT06737887.

## Conflicts of Interest

The authors declare no conflicts of interest.

## Supporting information


**Appendix S1:** SPIRIT Checklist for trials.

## Data Availability

Owing to ethical constraints, this study's data are not publicly available. They are available upon request from the corresponding author.
